# Special Issue: Synthesis, Processing, Structure and Properties of Polymer Materials

**DOI:** 10.3390/polym14214550

**Published:** 2022-10-27

**Authors:** Andrzej Puszka, Beata Podkościelna

**Affiliations:** Department of Polymer Chemistry, Faculty of Chemistry, Institute of Chemical Sciences, Maria Curie-Skłodowska University in Lublin, Gliniana 33, 20-614 Lublin, Poland

## 1. Introduction

Polymeric materials are widely used in many different technical fields. Modern polymers due to their properties are increasingly replacing traditional construction materials. Polymers and plastics that are non-flammable and resistant to very high temperatures, electrically conductive polymers, fully biodegradable plastics, shape memory polymers and self-repairing polymers are also known. Therefore they can be used to make elements of machines and mechanisms or can be applied as functional advanced materials. The development of polymeric materials is constantly being driven by increasing market demand.

Oil and gas processing produces polymers that are used to make inexpensive, very versatile plastics that make everyday life much easier. But as fossil fuels become scarcer, new challenges arise for the plastics and polymers market.

Innovative plastics are providing ever higher performance, but creation of new materials must be in line with environmental policy. Sustainable thinking is important at every stage, from the design, synthesis, processing and disposal of plastics after use. Researchers and industry are increasingly turning to waste-free and low-emission technologies. As a result, it is possible to obtain materials that are fully functional and exhibit good performance properties.

Due to the growing interest in modern polymeric materials, scientific information related to specialized literature on the complex chemical structure of plastics, as well as the possibility of modifying their features and properties during processing may be useful for specialists from various fields of science and technology.

The aim of this Special Issue was to highlight the progress and fundamental aspects for the synthesis, characterization, properties, and application of novel polymeric materials, as well as their copolymers, composites, and nanocomposites. The development of polymers is the greatest of all materials and the articles presented in our book are perfectly in line with the latest discoveries in this topic.

## 2. Short Description of the Articles presented in This Special Issue

In this Special Issue, 33 original articles have been published on various topics, including the preparation, characterization, and some examples of application of polymeric materials. This issue includes review articles [1,2,3,4,5,6,7,8] and research articles [9,10,11,12,13,14,15,16,17,18,19,20,21,22,23,24,25,26,27,28,29,30,31,32,33].

In the paper of Usri, Jamain and Makmud [1] methods for the synthesis of cyclotriphosphazene derivatives are outlined. The flame retardant and dielectric properties of various types of polymers doped with these compounds are also presented.

The relationship between the structure and properties of azo-compound-based liquid crystals was described by Razali and Jamain [2]. In the paper, they have demonstrated that few factors influenced on the formation of different liquid crystals: the length of the alkyl terminal chain, inter/intra-molecular interaction, presence of spacer, spacer length, polarization effects, odd-even effects, and the presence of an electron-withdrawing group or an electron-donating group.

Another 5 papers were devoted to organic-inorganic materials [3] and polymer composites [4,5,6,7]. In the review paper of Ikake, Hara and Shimizu [3], the authors discussed the organic-inorganic hybrid materials which have become indispensable high-performance and highly functional materials. This is owing to the improved dispersion control in hybrid materials and the emergence of functional ionic liquids. Harmonization of both these factors has enabled the utilization of functional 3D network structures and nanodispersions in composite materials. The authors summarized the historical development of hybrid materials prepared using the sol-gel method and the birth of ionic liquids.

In the paper of Mousavi et al. [4], summarized the recent advances in using bioactive Graphene Quantum Dots-based polymer composites in drug delivery, gene delivery, thermal therapy, thermodynamic therapy, bioimaging, tissue engineering, bioactive GQD synthesis, and GQD green resuscitation, in addition to examining GQD-based polymer composites.

The paper of Zaidi et al. [5] investigated the impact of carbon nanotubes influence on the properties of the polymer/CNT nanocomposies for photovoltaic application.

The paper of Ogunleye and Rusnakova [6], reviews some of the studies related to the techniques associated with fibre-prestressing in polymer matrix development. The reports from this study provide some basis for selecting a suitable technique for prestressing as well as measuring residual stresses in composite materials.

In the paper of Gómez-Gast et al. [7], the authors presents an overview of polyhydroxyalcanoate–vegetal fiber composites, the effects of the fiber type, and the production method’s impact on the mechanical, thermal, barrier properties, and biodegradability, all relevant for biopackaging.

In the last review paper of Liu an Claesson [8], the authors summarize recent experimental investigations of the interfacial lubrication properties of surfaces coated with bottlebrush bio-lubricants and bioinspired bottlebrush polymers. The authors also discuss recent advances in understanding intermolecular synergy in aqueous lubrication including natural and synthetic polymers. Finally, opportunities and challenges in developing efficient aqueous boundary lubrication systems are outlined.

The scope of research articles of papers included a Special Issue is very wide. It includes papers on the synthesis and study of the properties of new thermoplastic polymers [9,10,11,12,13], obtaining of composites [14,15,16], nanocomposites [17,18,19], microspheres [20,21], polymeric blends [22,23,24], hydrophobic nanostructures [25], polymeric membranes [26,27], hydrogels [28] and others [29,30,31,32,33].

In the paper of Hernández-Fernández et al. [9], the effects of arsine on the synthesis and thermal degradation of virgin polypropylene and proposes reaction mechanisms that allow understanding of its behaviour are studied.

In another paper, Wnuczek et al. [10] proposed the synthesis of novel polycarbonates obtained without the use of toxic bisphenol A.

In the next two articles, the authors presented a method of obtaining new polyurethanes. Gomez et al. [11] described the preparation and properties of bio-polyurethanes obtained from residual palm oil without and with jatropha oil or algae oil as additions. Such polymers have a high potential for the production of environmentally friendly bio-based polyurethanes. Puszka and Sikora [12] presented the synthesis and detailed characterization of the structure and properties of poly(thiourethane-urethane) elastomers with a polycarbonate soft segment.

The paper of Serra-Aguila et al. [13] examined the development of a procedure to predict tensile moduli at different strain rates and temperatures, using experimental data from three-point-bending dynamic mechanical analysis. The method was validated by means of a prediction of tensile moduli of polyamide PA66 in the linear elastic range, over a temperature range that included the glass-transition temperature.

Several and important components of the Special Issue were papers describing polymer composites as well as nanocomposites. Ahmad and Abdul Rahman [14], used an electrospinning technique to obtain nanofibers of lignin-filled polyacrylonitrile composites. Removal of lignin from the polymer matrix by selective dissolution yielded fibers with a porous structure was described in detail.

In the paper of Fouly et al. [15] the green composite from corn cob powder and epoxy resin was presented. For the materials obtained, they studied the mechanical and tribological properties, and compared the experimental results achieved with theoretical calculations.

In turn Wang [16] examined the effect of a halogen-free flame retardant on the kinetics of the thiol-ene reaction. The results indicated that flame retardant promoted cross-linking reactions, to form a compact char layer and retarded further the thermal degradation of the polymer matrix.

Díez-Rodríguez et al. [17] obtained poly(ε-caprolactone) composites with mesoporous SBA-15 nanosilica by extrusion. They studied the effect of the filler on the morphology, thermal and mechanical properties of the obtained compositions.

In the article of Merzah et al. [18], the authors presented the preparation of treated nanoclay composites with epoxy resin. The changes in mechanical and thermomechanical properties as well as morphology depending on the amount of filler in the composites were determined.

Chen et al. [19] used an in-situ polymerization method to prepare electrically conductive PANDB/γ-Al_2_O_3_ core-shell nanocomposites. For the resulting composites, the authors determined the changes in electrical conductivity and characterized the morphology and structure of the materials.

Wang’s and others’ paper [20] describes synthesis of novel crosslinked polyphosphazene-aromatic ether organic–inorganic hybrid microspheres with different structures. The materials were synthesized via precipitation polycondensation between hexachlorocyclotriphosphazene (HCCP) and bisphenol monomers. The changes in the structure, morphology and wettability of the films obtained by immersing a silicon wafer in ethanol-dispersed microspheres were studied.

In the paper of Rezić et al. [21], optimization of the microencapsulation process through two steps of simple and complex coacervation for further effective functionalization of polymers by dip-coating methodology was presented. The authors prepared functionalized different polymer surfaces with antimicrobial core-shell microcapsules and characterized of polymers after functionalization by several spectroscopic and microscopic techniques.

Masek et al. [22] have studied the influence of natural substances on the ageing properties of epoxidized natural rubber (ENR) and poly (lactic acid) (PLA) eco-friendly elastic blends. Additionally, the ENR/PLA blends were filled with natural pro-health substances of potentially antioxidative behavior, namely, δ-tocopherol (vitamin E), curcumin, δ-carotene and quercetin. Their finding paves new opportunities for bio-based and green anti-ageing systems employed in polymer technology.

In the paper of Lee et al. [23], high-density polyethylene (HDPE)/ethylene vinyl acetate (EVA)/polyurethane (PU) blends were prepared by radiation crosslinking to improve the thermal and mechanical properties of HDPE. This modification method is extremely important and can be applied to various polymer products requiring high heat resistance and flexibility, such as electric cables and industrial pipes.

The aim of the work by Chang et al. [24] was to obtain fully biodegradable polymer blends derived from thermoplastic starch and poly (butylene adipate-*co*-terephthalate). These blends were further foamed with carbon dioxide to improve the flexibility and reduce the brittleness of the finished materials. Due to the fact, that obtained materials exhibits improved flexibility, lightweight and environment-friendly nature, they could be used in electronics packaging materials and has medical equipment application potential in the future.

Hossain and Drmosh [25] reported a simple and inexpensive one-step process to fabricate polymer-templated hydrophobic nanostructures for H_2_ sensing application. As a polymer matrix, they used commercial polycarbonate which they subjected to appropriate chemical treatment and then deposited palladium particles on it.

In the paper of Li et al. [26], alkali-induced grafting proton exchange membranes with *co*-grafting PVDF with α-methylstyrene and acrylonitrile were reported. The effects of the types and contents of the alkalis and solvents on the grafting polymerization were investigated and the obtained PVDF-based proton exchange membranes were characterized in terms of the morphology, liquid uptake capability, swelling, ion exchange capacity, conductivity, mechanical property and so on.

In the paper of Rosiles-González et al. [27], the authors reported the synthesis of block and random copolymers of 2-acrylamido-2-methyl-1-propane sulfonic acid (AMPS) and methyl methacrylate (MMA), with different AMPS feed ratios. These solution-processable copolymers with strongly sulfonated acid groups resulted in membranes with tunable ion exchange and water absorption capacities.

Chi et al. [28] obtained and characterized novel metal-supramolecular hydrogels. The hydrophilic polymers were based on acrylic acid and maleic anhydride, and in addition, the polymer chain was grafted with terpyridine units. The supramolecular hydrogels with different terpyridine contents were prepared via the Ni^2+^–terpyridine coordination bonds formed in aqueous solution. Based on the results, the authors assume that such coordination hydrogels in the future can be used in environmental reactions, biosensors and the similar.

In the paper of Radwan et al. [29] are presented study of the mechanical properties of peat stabilized with different percentages of fillers (including PP fibers).

El-Sayed [30] described the preparation and degradation of geopolymer concrete samples during compression.

Pérez et al. [31], reported the effect of flocculation time, flocculant dose, and water quality on the shear strength of kaolin aggregates. The research contained in this work are useful for decision making in the design and operation of water thickeners and clarifiers.

In the paper of Lorente et al. [32], the relationship between the processing conditions of the solution blow spinning process used to prepare nonwoven mats of polyethylene oxide (PEO), and the structure and morphology of the resulting materials are studied. In this research, the influence of the solvent used to preparation the PEO solutions to be blow spun was considered. The authors concluded, that by changing the solvent composition used to dissolve, the different thermal, mechanical and dissolution behaviors of the material were found.

Pîrvu and Deleanu [33] have attempted to identify failure mechanisms and to measure a well-accepted characteristic, backface signature (BFS), for the designed ballistic protection package. The authors measured BFS and investigated failure mechanisms of this particular package in order to evaluate it as a future application in body armor.

## Data Availability

Not applicable.
